# Gut microbiota diversity and specific composition during immunotherapy in responders with non-small cell lung cancer

**DOI:** 10.3389/fmolb.2022.1040424

**Published:** 2022-10-24

**Authors:** Fumihiro Shoji, Masafumi Yamaguchi, Masaki Okamoto, Shinkichi Takamori, Koji Yamazaki, Tatsuro Okamoto, Yoshihiko Maehara

**Affiliations:** ^1^ Department of Thoracic Surgery, Clinical Research Institute, National Hospital Organization, Kyushu Medical Center, Fukuoka, Japan; ^2^ Department of Thoracic Oncology, National Hospital Organization, Kyushu Cancer Center, Fukuoka, Japan; ^3^ Department of Respiratory Medicine, Clinical Research Institute, National Hospital Organization, Kyushu Medical Center, Fukuoka, Japan; ^4^ Department of Surgery, Kyushu Central Hospital, Fukuoka, Japan

**Keywords:** oral and gut microbiota, diversity, specific composition, non-small cell lung cancer, immune checkpoint inhibitors response

## Abstract

Cancer immunotherapy including immune checkpoint inhibitors (ICI) has revolutionized non-small cell lung cancer (NSCLC) therapy. Recently, the microbiota status “before” initiation of ICI therapy has been emphasized as a predictive biomarker in patients undergoing ICI therapy. However, the microbiota diversity and composition “during” ICI therapy is unknown. This multicenter, prospective observational study analyzed both saliva and feces from 28 patients with NSCLC. We performed 16S ribosomal RNA gene sequencing, then analyzed associations of oral and gut microbiota diversity or composition with ICI response. At the genus level, the alpha diversity of the gut microbiota was significantly greater in responders (*n* = 17) than in non-responders (*n* = 11) (Chao 1, *p* = 0.0174; PD whole tree, *p* = 0.0219; observed species, *p* = 0.0238; Shannon, *p* = 0.0362), while the beta diversity of the gut microbiota was significantly different (principal coordinates analysis, *p* = 0.035). Compositional differences in the gut microbiota were observed between the two groups; in particular, g_*Blautia* was enriched in responders, whereas o_*RF32 order unclassified* was enriched in non-responders. The progression-free survival (PFS) of patients enriched gut microbiota of g_*Blautia* was significantly longer [median survival time (MST): not reached vs. 549 days, *p* = 0.0480] and the PFS of patients with gut microbiota of o_*RF32 unclassified* was significantly shorter (MST: 49 vs. 757 days, *p* = 0.0205). There were no significant differences between groups in the oral microbiota. This study revealed a strong association between gut microbiota diversity and ICI response in NSCLC patients. Moreover, specific gut microbiota compositions may influence the ICI response. These findings might be useful in identifying biomarkers to predict ICI response.

## Introduction

Immunotherapy with immune checkpoint inhibitors (ICI) is widely used to treat various malignancies, including non-small cell lung cancer (NSCLC); it has revolutionized therapeutic approaches to cancer. Programmed death-ligand-1 (PD-L1) is an immune checkpoint protein expressed on tumor cells and tumor-infiltrating immune cells, which can mediate anticancer immunosuppression (Ribas and Wolchok, 2018). Anti-PD-1 antibodies (e.g., nivolumab and pembrolizumab) and anti-PD-L1 antibodies (e.g., atezolizumab and durvalumab) enable T-cell activation and immune system recognition.

Although tumorous PD-L1 expression is a potential biomarker of the ICI therapeutic response, there is no widely accepted optimal biomarker to predict the efficacy of ICI, because ICI response and survival outcomes show heterogeneity in NSCLC patients receiving ICI therapy, regardless of PD-L1 expression level (Rittmeyer et al., 2017; Gandhi et al., 2018; Paz-Ares et al., 2018; Socinski et al., 2018).

We recently reported that the pretreatment host immune-nutritional condition was a prognostic marker for NSCLC patients receiving ICI therapy (Shoji et al., 2019). Host immunity is clearly associated with the ICI response. The internal microbiome is regarded as a controlling factor in host immunity. In particular, the gut microbiome can modulate the host immune response (e.g., anti-tumor immunity) and optimize both innate and adaptive immune responses (Littman, 2012). Recently, preclinical studies have shown that the gut microbiome composition and its modification in murine models could influence the efficacy of ICI (Corrales et al., 2015; Vétizou M, 2015). Therefore, the microbiome has been emphasized as a predictive biomarker of ICI therapy, mainly in studies from the United States or Europe. Additionally, the gut microbiota diversity or abundance of specific gut microbiome components has been correlated with the efficacy of anti-PD-1 antibody in melanoma patients (Fessler et al., 2018). Moreover, fecal microbiota transplantation (FMT) in murine models might restore the ICI response (Gopalakrishnan et al., 2018; Routy et al., 2018). In a recent study, FMT from ICI responders to ICI non-responders produced ICI efficacy in melanoma patients (Davar et al., 2021).

Furthermore, the oral microbiota has been associated with several diseases (e.g., inflammatory bowel disease and allergic diseases) through its influence on the gut microbiota (Pietrantoni et al., 2003; Yamasaki et al., 2013; Atarashi K, 2017). A recent study revealed that variation in the oral microbiota was associated with a risk of lung cancer (Hosgood et al., 2021). However, samples were collected prior to ICI therapy in most previous studies, and thus minimal information has been available regarding the microbiota status during ICI therapy. Accordingly, FMT or biotics therapy approaches are needed to investigate changes in the microbiota during ICI therapy. Notably, there are definite differences in microbiota composition among ethnicities (Nishijima et al., 2016); to the best of our knowledge, few reports have been published regarding Japanese NSCLC patients (Hakozaki et al., 2020). In addition, the present study is based on the clinical question how is the condition of host microbiome in NSCLC patients during ICI therapy. Therefore, the present study might be meaningful as one of the pioneer studies highlighted the microbiome status during ICI therapy.

Here, we performed a prospective study to clarify the microbiota diversity and composition in Japanese NSCLC patients by analyzing samples collected during ICI therapy.

## Materials and methods

### Study design and participants

This prospective observational study was conducted at multiple centers: Department of Thoracic Surgery and Department of Respiratory Medicine, Clinical Research Institute, National Hospital Organization (NHO) Kyushu Medical Center and Department of Thoracic Oncology, NHO Kyushu Cancer Center. Eligibility criteria were as follows: pathologically or cytologically confirmed diagnosis of locally advanced/unresectable or postoperative recurrent NSCLC; receipt of ICI monotherapy including nivolumab (Opdivo, Bristol-Myers Squibb), pembrolizumab (Keytruda, Merck), atezolizumab (Tecentriq, Genentech), and durvalumab (Imfinzi, Astra Zeneca) or platinum-based therapy combined with these ICI agents. Patients were also enrolled if they had discontinued these therapies but had not received any additional therapies. Patients with the presence of ongoing antibiotics therapy for infectious diseases before/during ICI therapy, were excluded in the present study.

From July 2019 to December 2020, 34 NSCLC patients were eligible and enrolled. Of those 34 patients, 28 had both saliva and feces samples available for this study (Supplementary Figure S1). All enrolled patients had at least one measurable target lesion based on the Response Evaluation Criteria in Solid Tumors (RECIST), version 1.1 (Eisenhauer et al., 2009). Clinical/pathological stage was based on the Tumor Node Metastasis (TNM) classification established by the International Union Against Cancer (Goldstraw et al., 2016). For TNM staging, all patients underwent computed tomography (CT) of the thorax and upper abdomen, as well as bone scintigrams, brain CT scans, magnetic resonance imaging (MRI), or fluorodeoxyglucose-positron emission tomography (FDG-PET). Postoperative local or distant recurrence was defined as described previously (Varlotto et al., 2009). ICI therapy was continued until radiographic progression. PD-L1 protein expression was evaluated using antibody clone 22C3 (Dako, Agilent Technologies, Santa Clara, CA, United States).

### Sample collection

**FIGURE 1:**
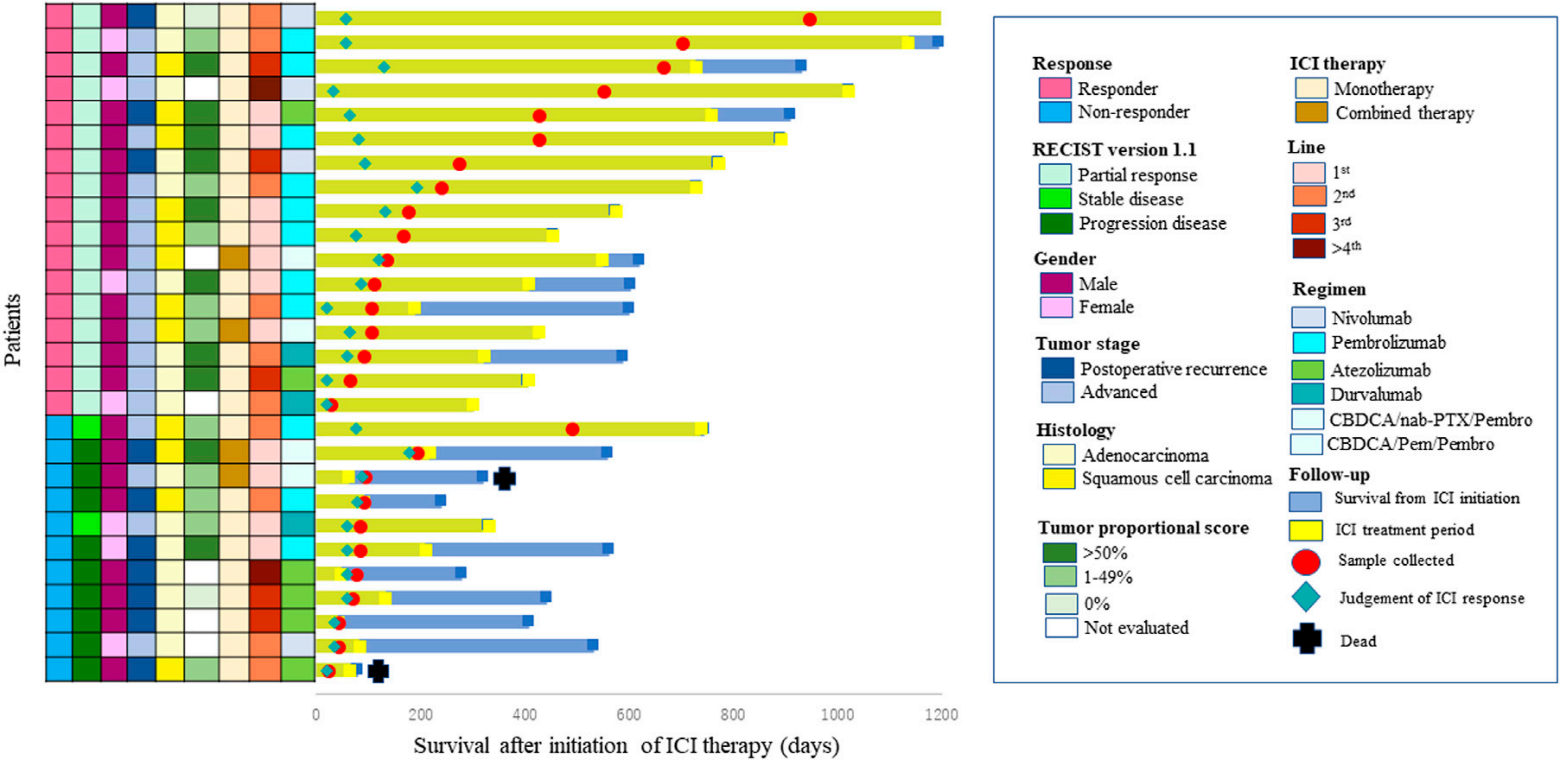
Swimmer plot of survival after initiation of immune check point inhibitor (ICI) therapy. Each bar represents one patient. The left column shows clinicopathological characteristics.

Salivary and fecal samples were collected in sterile containers and immediately placed at 4°C, then frozen at −80°C. Individual periods of sample collection are shown in Figure 1. The mean numbers of days between the initiation of ICI therapy and the day of sample collection were 307 (29–945) days in ICI responders and 117 (23–491) days in ICI non-responders.

### DNA extraction, gene amplification, sequencing, and data analysis procedures

Preliminary treatment of fecal samples was conducted in accordance with a previously described method (Takahashi et al., 2014); DNA was then extracted using an automated DNA isolation system (Gene Prep Star PI-480, Kurabo, Japan). DNA was extracted from saliva using the Mora-Extract kit (Kyokuto Pharmaceutical, Japan). The V3–V4 regions of bacterial 16S rRNA genes were amplified using the Pro341F/Pro805R primers (Takahashi et al., 2014) and dual-index method (Hisada et al., 2015) under hemi-nested PCR conditions (Hell et al., 2013). Barcoded amplicons were paired-end sequenced on a 2 × 284-bp cycle using the MiSeq system with MiSeq Reagent Kit chemistry, version 3 (600 Cycle). Paired-end sequencing reads were merged using the fastq-join program with default settings (Aronesty, 2013). Only reads with quality value (QV) scores of ≥ 33 were extracted with split_libraries_fastq.py command in QIIME, version 1.8.0 (Caporaso et al., 2010). Chimeric sequences were removed using USEARCH61 (Edgar et al., 2011) with the identify_chimeric_seqs.py command in QIIME (Caporaso et al., 2010). Operational taxonomic units (OTUs) were aligned using the pick_open_reference_otus.py command in QIIME (Caporaso et al., 2010). OTUs with 97% similarity were identified with the Greengenes database, version 13.8 (Aitchison, 1986). Alpha diversity indices (e.g., observed species, Chao-1, Shannon, and PD_whole_tree) and beta diversity indices [e.g., principal coordinates analysis (PCoA)] were analyzed using the alpha_rarefaction.py and beta_diversity.py commands in QIIME, respectively (Edgar et al., 2011). Sampling depth in alpha and beta diversity was 50,895, which was the minimum number of read counts among samples. To account for compositional artifacts, we transformed relative abundances using the Centered Log-Ratio (CLR) transformation (Aitchison, 1986). The Chao-1 index was used to determine community richness and the Shannon index was used to determine community diversity. The PD_whole_tree index was used to compute Faith’s phylogenetic diversity. PCoA was used to show differences between the two groups. Unweighted UniFrac metrics were used for beta diversity (DeSantis et al., 2006). In order to identify the distinct gut microbiota between ICI responders and non-responders, the Linear Discriminative Analysis Effect Size (LEfSe) method was used to compare the composition of the gut microbiota in genus level using an online tool (http://www.ehbio.com/ImageGP/index.php/Home/Index/LEFSe.html).

### Statistical analysis

Categorical variables were analyzed using Fisher’s exact test. Continuous variables were compared using the chi-squared test. The Mann-Whitney *U* test was used to determine significant differences among the different groups using alpha diversity, which showed the diversity in each individual sample. Logistic regression analysis to calculate odds ratios for ICI response with respect to clinic-pathological characteristics was used. Kaplan-Meier statistics and log-rank testing to evaluate progression-free survival (PFS) was applied.

Statistical analyses were performed using JMP software, version 14.0 (SAS Institute, Inc., Cary, NC, United States). The adonis function in the vegan package of R software, version 3.6.1, was used to conduct permutational multivariate analysis of variance (PERMANOVA) with respect to microbiome composition. *p-*values < 0.05 were considered statistically significant.

## Results

### Patient characteristics

The results were determined in follow-up examinations over a mean duration of 598 days (range, 81–1,225 days) after initial ICI therapy. Patient characteristics are shown in Supplementary Table S1. The study group included seven women and 21 men, with a mean age at diagnosis of 71 years (range, 56–88 years). Fifteen patients (53.6%) had ECOG performance status (PS) 0 and 13 (46.4%) had ECOG-PS 1. Seven patients (25.0%) had never smoked, and the remaining 21 patients were current or former smokers. The histological types were adenocarcinoma in 16 patients (57.1%) and squamous cell carcinoma in 12 patients (42.9%). Of the 28 included patients, one (3.6%) had stage IIA, seven (25.0%) had unresectable stage III (two with IIIA, three with IIIB, and two with IIIC), 11 (39.3%) had stage IV (seven with IVA and four with IVB), and nine (32.1%) had postoperative recurrence. Seven patients (25.0%) had mutant epithelial growth factor receptor (EGFR) and 21 patients (75.0%) had wild-type EGFR or no data regarding EGFR status. ICI was first-line therapy in 11 patients (39.3%), second-line therapy in 10 patients (35.7%), third-line therapy in five patients (17.8%), and fourth-line or later therapy in two patients (7.2%). Twenty-four patients (85.7%) received ICI monotherapy (nivolumab: 3 mg/kg or 240 mg/body intravenously at 2-week intervals; pembrolizumab: 200 mg/body intravenously at 3-week intervals; atezolizumab: 1,200 mg/body intravenously at 3-week intervals; or durvalumab: 10 mg/kg intravenously at 2-week intervals), while the remaining four patients received ICI therapy combined with platinum-doublet chemotherapy [pembrolizumab: 200 mg/body plus carboplatin, area under the curve (AUC) for concentration-time: 5 mg/ml/minute; plus pemetrexed 500 mg/m^2^ or nab-paclitaxel 200 mg/m^2^ intravenously at 3-week intervals]. Ten patients (35.7%) had more than 50% tumorous PD-L1 expression, 10 patients (35.7%) had 1%–49% PD-L1 expression, and eight patients (28.6%) had no PD-L1 expression or no data regarding PD-L1 status.

### Immune checkpoint inhibitors response in non-small cell lung cancer patients

No patient experienced a complete response (CR) (0%), 17 patients had a partial response (PR) (60.7%), two patients had stable disease (SD) (7.2%), and nine patients (32.1%) had progressive disease (PD). Therefore, 17 patients (PR) were regarded as ICI responders; the remaining 11 patients (SD or PD) were regarded as ICI non-responders (Supplementary Figure S1). Logistic regression analysis to calculate odds ratios for ICI response with respect to clinic-pathological characteristics such as gender, smoking status, number of prior systemic therapy, regimen, histology and tumor proportional score, was performed (Supplementary Table S2). As a result, there was no significant difference between ICI response and any clinic-pathological features in multivariate analysis.

### Relative abundances in oral and gut microbiomes

**FIGURE 2:**
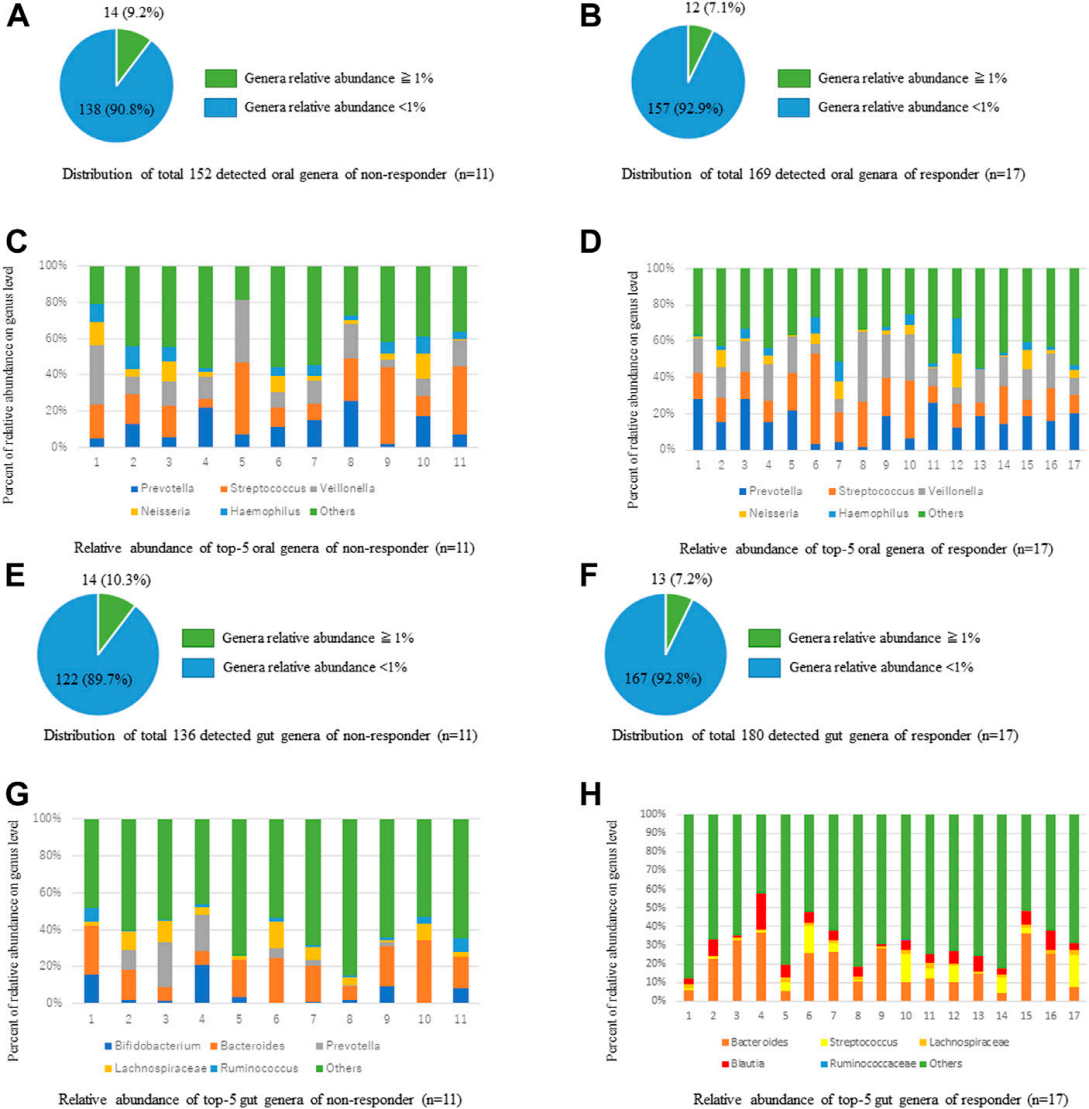
Comparisons of taxa relative abundances between ICI responders and non-responders at genus level. **(A,B)** Distributions of oral microbiota taxa relative abundances > 1% and < 1% in ICI non-responders **(A)** and responders **(B)**. **(C,D)** Comparisons of oral microbiota taxa relative abundances in ICI non-responders **(C)** and responders **(D)**. **(E,F)** Distributions of gut microbiota taxa relative abundances > 1 % and < 1% in ICI non-responders **(E)** and responders **(F)**. **(G,H)** Comparisons of gut microbiota taxa relative abundances in ICI non-responders **(G)** and responders **(H)**.

We analyzed the relative abundances of oral bacteria at the phylum and genus levels. At the phylum level, p_*Firmicutes*, p_*Bacteroides*, p_*Proteobacteria*, p_*Actinobacteria*, p_*Fusobacteria*, and p_*TM 7* were the main taxa; these taxa comprised more than 99% in all groups. At the genus level, 169 genera were detected in the ICI responder saliva microbiota, while 152 species were detected in the ICI non-responder saliva microbiota (Figures 2A,B). Among the genus taxa with < 1% relative abundance, 157 genera (92.9%) were identified in the ICI responder saliva microbiota, while 138 genera (90.8%) were identified in the ICI non-responder saliva microbiota. Genera in both groups mainly included g_*Streptococcus*, g_*Veillonella*, g_*Prevotella*, g_*Haemophilus*, and g_*Neisseria* (Figures 2C,D). Regarding the gut microbiota, at the phylum level, p_*Firmicutes*, p_*Bacteroides*, p_*Actinobacteria*, p_*Proteobacteria*, p_*Fusobacteria*, and p_*Verrucomicrobia* were the main taxa (comprising more than 99%) in ICI responders. p_*Firmicutes*, p_*Bacteroides*, p_*Actinobacteria*, p_*Proteobacteria* and p_*Fusobacteria* were the main taxa (comprising more than 99%) in ICI non-responders. At the genus level, 180 genera were detected in the ICI responder gut microbiota, while 136 genera were detected in the ICI non-responder gut microbiota (Figures 2E,F). Among the genus taxa with < 1% relative abundance, 167 genera (92.8%) were identified in the ICI responder gut microbiota, while 122 genera (89.7%) were identified in the ICI non-responder gut microbiota. Genera in ICI responders mainly included g_*Bacteroides*, f_*Ruminococcaceae;* g_, f_*Lachnospiraceae;* g_, g_*Streptococcus*, and g_*Blautia*; genera in ICI non-responders mainly included g_*Bacteroides*, f_*Ruminococcaceae;* g_, f_*Lachnospiraceae;* g_, g_*Prevotella*, and g_*Bifidobacterium* (Figures 2G,H).

### Diversity metrics in oral and gut microbiomes

**FIGURE 3:**
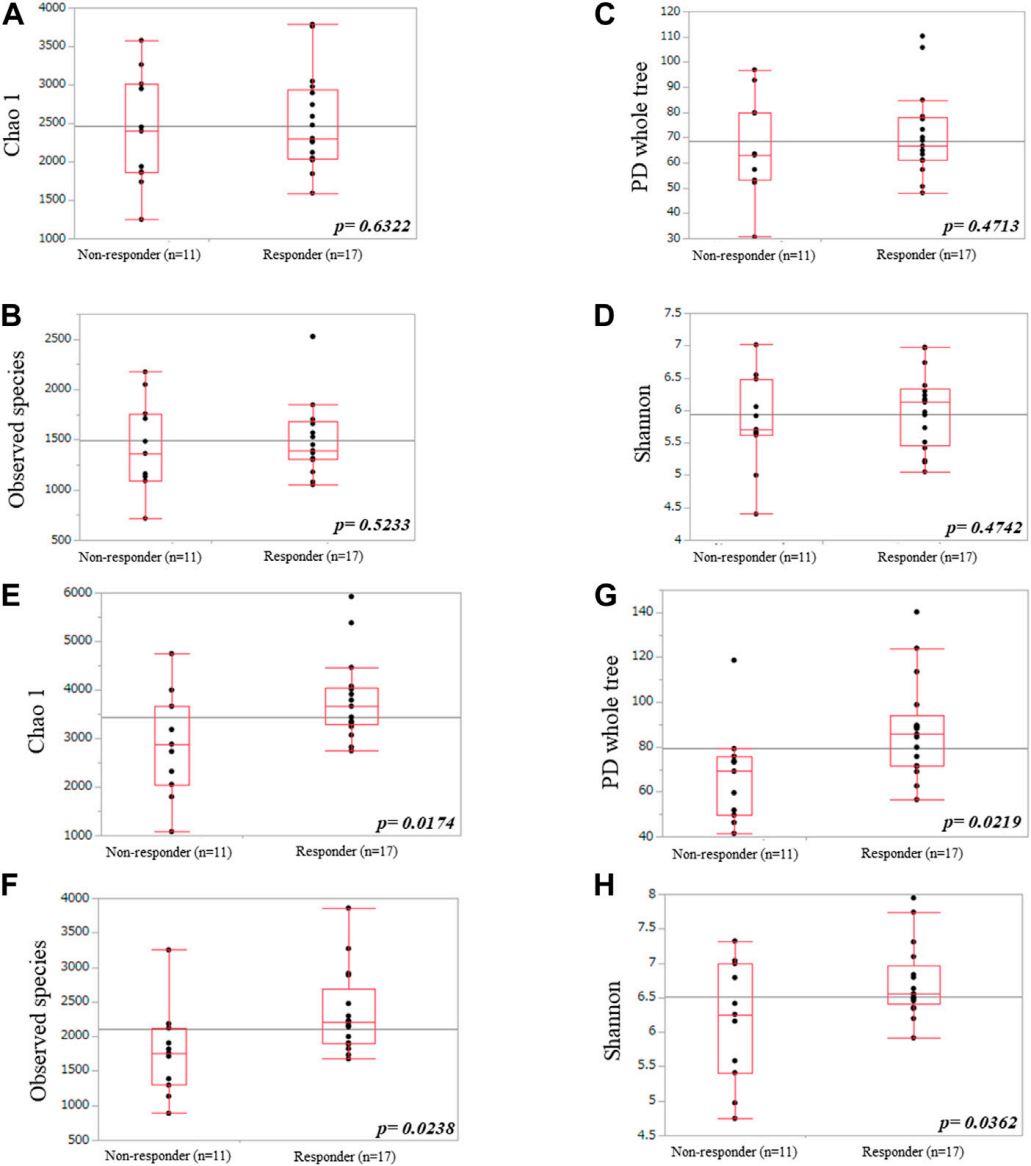
Comparisons of oral **(A–D)** and gut **(E–H)** microbiota diversities between ICI non-responders and responders. **(A,E)** Chao1 index, **(B,F)** observed species, **(C,G)** PD_whole_tree, and **(D,H)** Shannon index.

**FIGURE 4:**
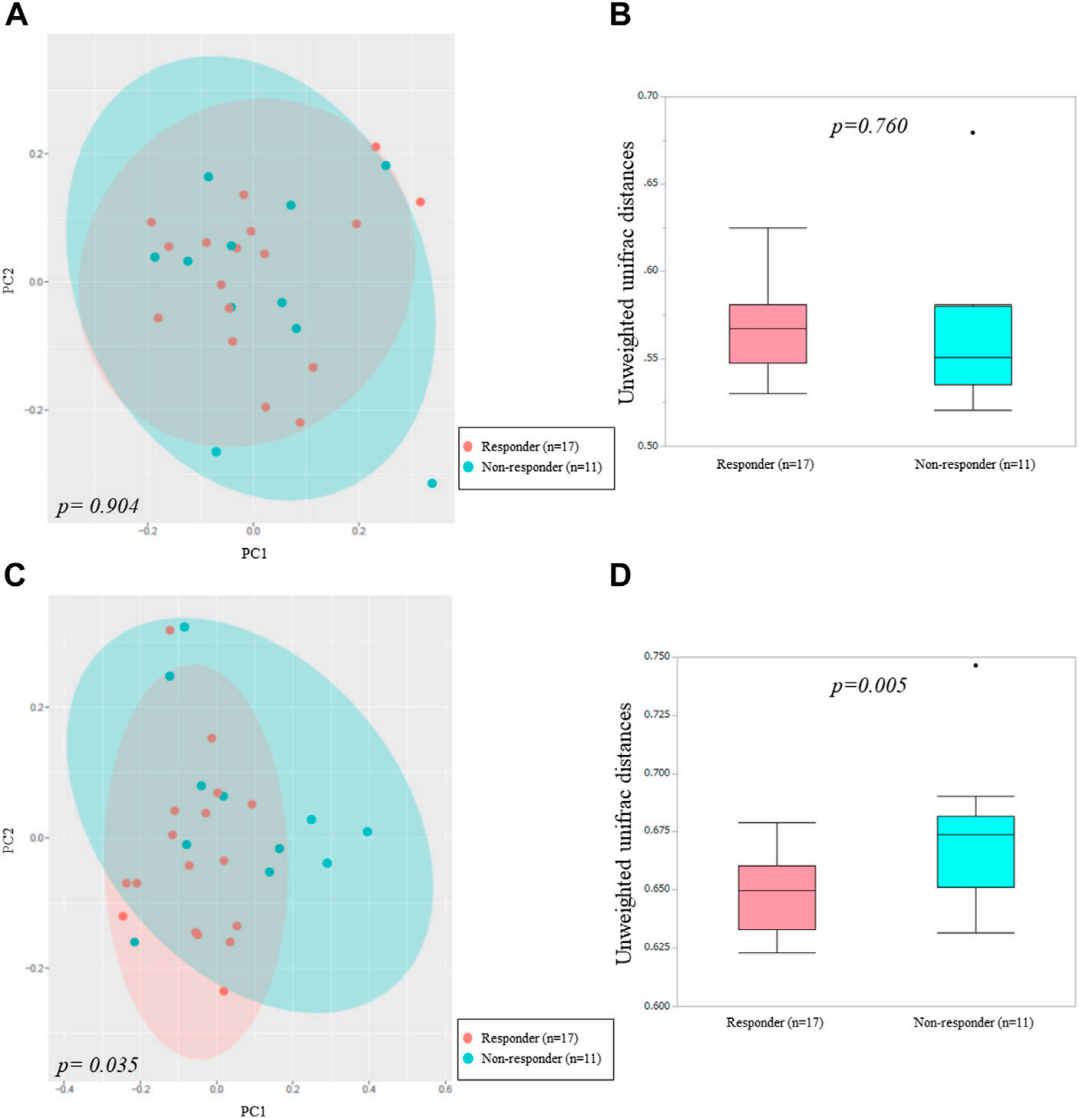
Principal coordinates analysis **(A,C)** and boxplots **(B,D)** of microbiota data based on unweighted UniFrac distances between ICI non-responders and responders at the genus level.

We used alpha and beta diversity indices to evaluate the intersample and intrasample relationships in the oral and gut microbiota. Figures 3, 4 show the alpha diversity metrics of oral and gut microbiota at the genus level. In the oral microbiota, there were no significant differences between the two groups (Chao 1, *p* = 0.632; Observed species, *p* = 0.523; PD_whole_tree, *p* = 0.471; and Shannon, *p* = 0.474) (Figures 3A–D). In the gut microbiota, all alpha diversity metrics were significantly higher in ICI responders than in ICI non-responders (Chao 1, *p* = 0.017; Observed species, *p* = 0.024; PD_whole_tree, *p* = 0.022; and Shannon, *p* = 0.036) (Figures 3E–H). PCoA assessment of beta diversity was conducted based on weighted UniFrac distance (Figure 4). In both oral and gut microbiota, similar patterns were evident in the two groups. The greatest variations in the oral and gut microbiota of the two groups were 4.39% (PC1) and 4.32% (PC2), and 4.51% (PC1) and 4.22% (PC2), respectively. PERMANOVA based on unweighted UniFrac distance confirmed significant differences between the two groups in the gut microbiota alone (Figure 4A, oral: *p* = 0.904 and Figure 4C, gut: *p* = 0.035). Additionally, The Mann-Whitney *U* test showed significant differences in the gut microbiota alone (Figure 4B, oral: *p* = 0.760 and Figure 4D, gut: *p* = 0.005).

### Linear discriminative analysis effect size results

**FIGURE 5:**
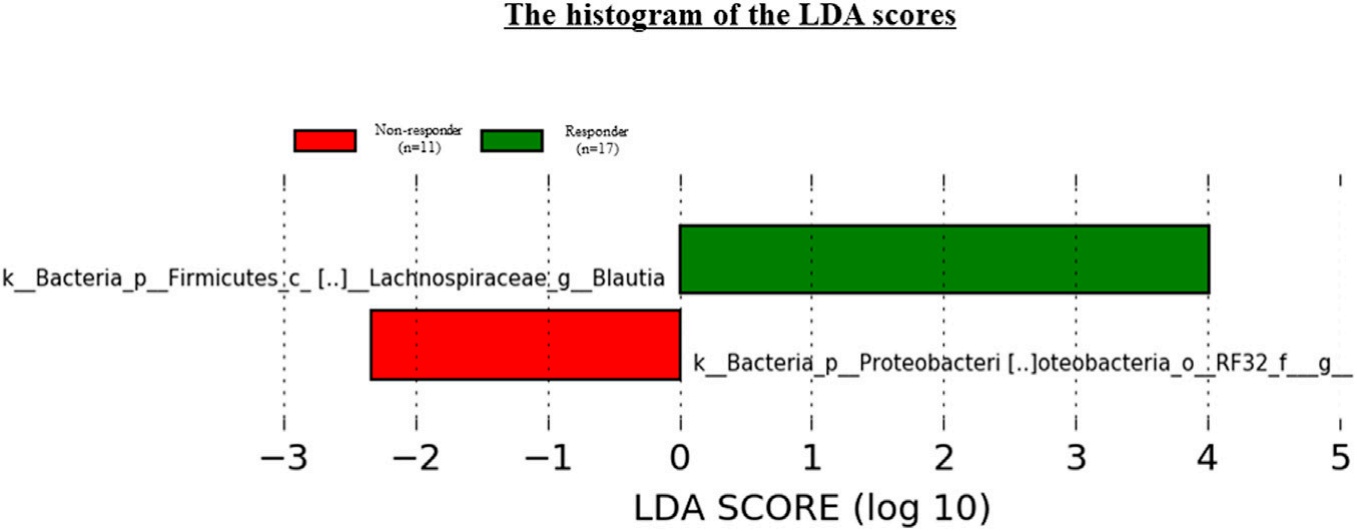
Results of LEfSe analysis of differentially abundant taxa in gut microbiota between ICI responders and non-responders.

We used LEfSe to perform high-dimensional genus comparisons regarding oral and gut microbiota between ICI responders and non-responders. Figure 5 shows that the ICI responder gut microbiota was significantly enriched for g_*Blautia*, compared with the ICI non-responder gut microbiota. In contrast, the ICI non-responder gut microbiota was significantly enriched for o_*RF32 unclassified*, compared with the ICI responder gut microbiota. There were no significant differences in the oral microbiota between the two groups.

### Analyses for progression-free survival and correlation between the microbiome and immune adverse events

**FIGURE 6:**
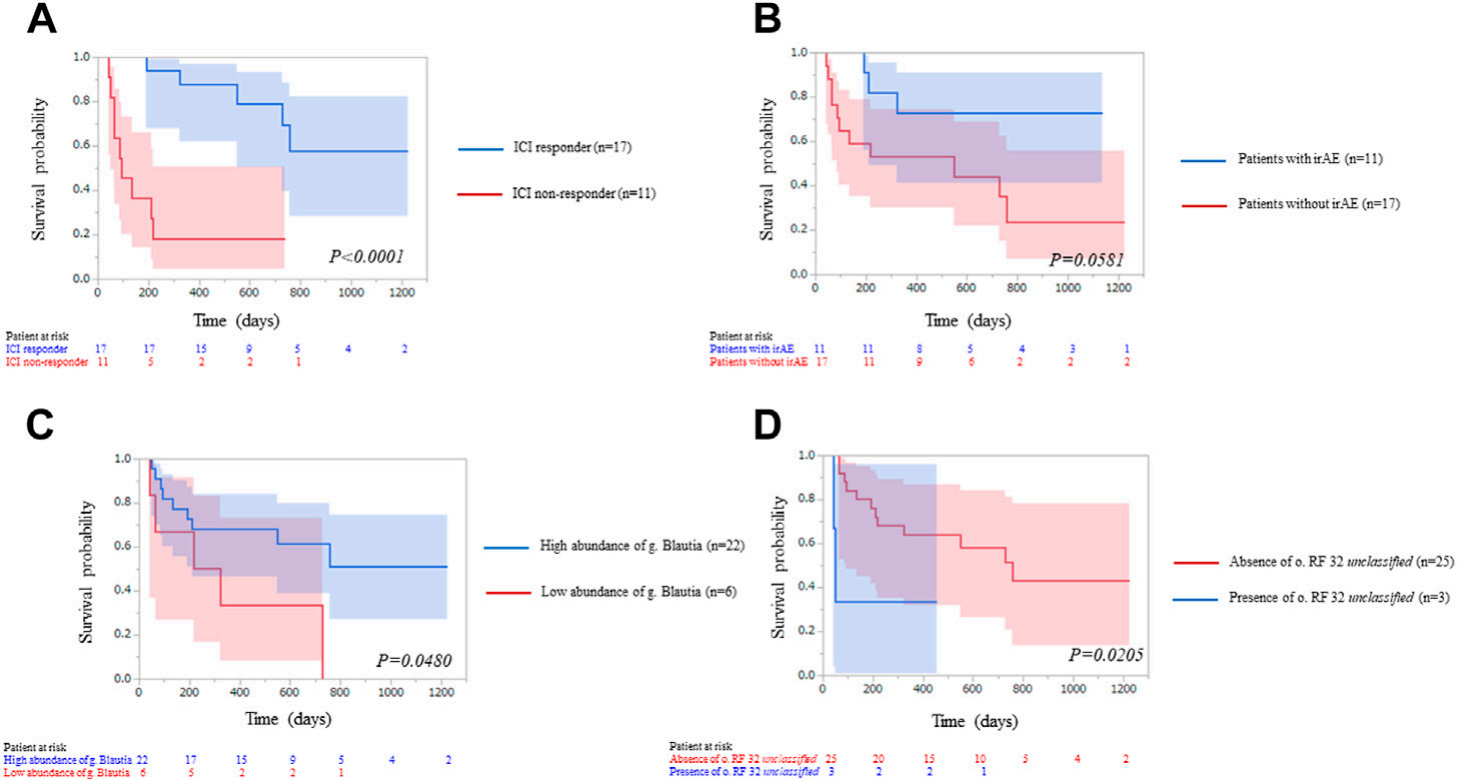
Kaplan–Meier curve analysis of progression free survival for 28 patients treated with immune-check point inhibitors (ICI) therapy by **(A)** ICI response, blue line: ICI responders; red line: ICI non-responders. The two groups were significantly different [Median survival time (MST): not reached vs. 92 days, *p* < 0.0001], **(B)** immune-related adverse event (irAE), blue line: patients with irAE; red line: patients without irAE. MST: not reached vs. 549 days, *p* = 0.0581), **(C)** abundance of g_*Blautia*, blue line: patients enriched gut microbiota of g_*Blautia*; red line: patients reduced gut microbiota of g_*Blautia*. The two groups were significantly different (MST: not reached vs. 549 days, *p* = 0.0480) and **(D)** presence of o. *RF32 unclassified*, blue line: patients with gut microbiota of o. *RF32 unclassified*; red line: patients without gut microbiota of o. *RF32 unclassified*. The two groups were significantly different (MST: 49 days vs. 757 days, *p* = 0.0205).

During a median follow-up of 598 days, 9 of 28 patients had immune-related adverse events (irAE). Six patients had hypothyroidism, 2 interstitial pneumonitis, 1 thyroiditis, 1 colitis and 1 hypoadrenalism. We applied Kaplan-Meier statistics and log-rank testing to evaluate PFS by ICI response, irAE, relative abundance of g. *Blautia* and o. *RF32 unclassified* (Figure 6). Median PFS was not reached in ICI responders, but median PFS was 92 days for non-responders (*p* < 0.0001). Median PFS was not reached in patients with irAE, but median PFS was 549 days for patients without irAE (*p* = 0.0581). Median PFS was not reached in patients enriched gut microbiota of g. *Blautia*, but median PFS was 549 days for patients reduced gut microbiota of g. *Blautia* (*p* = 0.0480). Median PFS was 49 days in patients with gut microbiota of o. *RF32 unclassified*, but median PFS was 757 days for patients without gut microbiota of o. *RF32 unclassified* (*p* = 0.0205).

## Discussion

Similar studies regarding ICI therapy have focused on the microbiome in patients who have not yet received ICI therapy. Those studies revealed that the microbiota diversity and composition before ICI therapy was a predictive biomarker for ICI response. Although variations in gut microbiota composition were observed in the previous studies, there has been minimal information regarding gut microbiota status during ICI therapy. This information is important for efforts to enhance ICI therapy through biotics therapy (e.g., pre-, pro-, and synbiotics) and/or FMT. This analysis of oral and gut microbiota profiles in Japanese NSCLC patients during ICI therapy produced several novel findings.

The gut microbiota might have an important role in the ICI response in NSCLC patients, although the oral microbiota conveyed information distinct from the gut microbiota. Greater numbers of gut microbiota species were observed in ICI responders than in ICI non-responders. Additionally, those microbiota mainly consisted of minor species (<1%) at the genus level. Moreover, the fourth and fifth majority of gut microbiota species in ICI responder were differed from that in ICI non-responder, while first to fifth majority of oral microbiota were same between two groups.

An important tool for objective evaluation of the above data involves analysis of microbiota diversity: the numbers or abundances of microorganisms colonizing the gut. Greater alpha diversity indicates larger numbers of species in the gut, which implies a distinct gut microbiota composition. Several studies have reported that the ICI response was influenced by the alpha diversity of the gut microbiota before ICI therapy (Hamady and Knight, 2007; Gopalakrishnan et al., 2018). In the present study, high alpha diversity was observed in the ICI responder gut microbiota. Thus, our results indicated that ICI responders had more abundant gut microbiota, compared with ICI non-responders, during ICI therapy.

Our study also revealed a significant association between beta diversity and ICI response. Higher beta diversity indicates a significant difference in gut microbiota composition between two samples. In this study, the intersample distance was significantly shorter in ICI responders than in ICI non-responders. Therefore, the ICI responder gut microbiota had significant similarity, compared with the ICI non-responder gut microbiota, which implies a simple approach to control the gut microbiota by adding biotics therapy to ICI therapy.

Additionally, we identified specific gut microbiota species in Japanese NSCLC patients receiving ICI therapy by using LEfSe. The results indicated that g_*Blautia* was enriched in ICI responders, whereas o_*RF32 unclassified* was enriched in ICI non-responders. Several studies have shown significant associations between various gut microbiota components [e.g., *Bifidobacterium longum* (Fessler et al., 2018), *Collinsella aerofaciens* (Fessler et al., 2018), *Enterococcus faecium* (Fessler et al., 2018), *Akkermansia muciniphila* (Routy et al., 2018), *Ruminococcaceae* (Gopalakrishnan et al., 2018), *Faecalibacterium genii* (Jin et al., 2019) and *Firmicutes* (Froehlich et al., 2017)] and clinical response to ICI therapy. These results differed among individual studies, including the present study, presumably due to factors such as patient disease (e.g., melanoma, renal cell carcinoma, and NSCLC) and ICI regimen. Indeed, Chaput et al. (2017) indicated that the gut microbiota composition depended on the ICI regimen. Moreover, these differences might be caused by ethnicities. Nishijima et al. (2016) compared the gut microbiota between the Japanese population and individuals from 11 other nations. Notably, the Japanese gut microbiota was considerably different from the microbiota of other populations; it was characterized by the highest abundances of *Blautia*, *Bifidobacterium*, *Collinsella*, *Streptococcus*, and an unclassified *Clostridiales* genus, compared with the microbiota from the remaining 11 countries.

In addition to its status as a species characteristic of the gut microbiota in Japanese individuals, *Blautia coccoides* is regarded as an effective probiotic species. *B. coccoides* is an anaerobe and Gram-positive species found in human fecal samples. Reduced numbers of *B. coccoides* are associated with several benign diseases (e.g., hepatic cirrhosis and encephalopathy, irritable bowel syndrome, acute diarrhea, idiopathic inflammatory bowel disease, intestinal inflammation, and diabetes mellitus) and some malignancies (e.g., colorectal and breast cancers) (Ghanem et al., 2012; Liu and Tong, 2012). Furthermore, some reports have revealed that increased numbers of *B. coccoides* might be beneficial for human health. *B. coccoides* is increased among individuals with diets high in resistant starch and arabinoxylan (Nielsen et al., 2014); moreover, it can reduce NF-κB activity in human colon cancer cells (Tap et al., 2011). Additionally, *Blautia obeum* is a gut microbiota component involved in the transformation of carcinogenic heterocyclic amines, and reduced abundance of this species may increase heterocyclic amine-induced colorectal cancer risk (Ocvirk et al., 2019). Myles et al. (2014) reported that high omega-3 intake altered colonic inflammation and increased *Blautia* abundance in a murine model. Recently, Martini et al. (2022) reported that *Blautia* species could be a potential biomarker of outcome in metastatic colorectal cancer and non-small cell lung cancer patients treated with combined ICI therapy. Therefore, *Blautia* may be a key gut microbiota component involved in the ICI response in Japanese NSCLC patients. Conversely, the relative abundance of *RF32 unclassified* has been positively correlated with colonic damage and inflammation (Castro-Mejía et al., 2016), which are presumably negative influences on immunity in NSCLC patients.

The findings in this study indicate that controlling both gut microbiome diversity and the abundances of specific gut microbiome species during ICI therapy might lead to ICI response enhancement in Japanese NSCLC patients. Future therapies targeting the gut microbiome by means of pre-, pro-, or synbiotics to enhance the ICI response might be considered from our findings.

This study had the following limitations. First, only a single sample collection was performed. Thus, it was unclear how the microbiota diversity and composition might have changed before and after ICI therapy. Second, the sample size was small, which may have interfered with meaningful conclusions. Total 28 patients consisted of 18 with advanced stage and 10 with postoperative recurrence. Therefore, a statistical power was insufficient to analyze patients with each stages separately. Third, the delay between microbiota analysis and start of ICI therapy was almost 3 times longer in responders versus non responders, which might have any modifying effect on microbiota alpha and beta-diversity. Lastly, this study allowed various ICI regimens with or without combination chemotherapy. In the future, we plan to perform a large, multicenter, prospective observational study to evaluate the association between ICI response and changes in gut microbiota by collecting samples at multiple points (before and during ICI therapy) for NSCLC patients receiving a specific ICI regimen.

In conclusion, this study revealed a strong association between gut microbiota diversity and ICI response in Japanese NSCLC patients. Moreover, specific gut microbiota compositions may influence the ICI response. These findings might be useful in identifying biomarkers to predict ICI response, as well as in developing biotic therapies to enhance the ICI response.

## Data Availability

The datasets presented in this study can be found in online repositories. The names of the repository/repositories and accession number(s) can be found in the article/Supplementary Material.

## Supplementary material

SUPPLEMENTARY FIGURE S1 Study flow chart.

## Abbreviations

CR, complete response; ECOG, European Clinical Oncology Group; EGFR, epidermal growth factor receptor; FMT, fecal microbiome transplantation; ICI, immune-check point inhibitors; irAE, immune-related adverse events; MST, median survival time; NSCLC, non-small cell lung cancer; PD, progressive disease; PD-L1, programmed death-ligand 1; PFS, progression free survival; PS, performance status; PR, partial response; SD, stable disease.
