# Supplementary material for: Gut microbiota diversity and specific composition during immunotherapy in responders with non-small cell lung cancer
Source: Front Mol Biosci. 2022 Oct 24;9:1040424. doi: 10.3389/fmolb.2022.1040424 (PMC9638091; doi:10.3389/fmolb.2022.1040424)
Supplement: Supplementary file 1 [file Table1.DOCX]

**Supplementary table 1**. Patient characteristics

| Items |  | No. (%) or Median (Range) |
| --- | --- | --- |
| Gender | **Male/female** | 21 (75.0)/7 (25.0) |
| Age | **Year** | 71 (56-88) |
| ECOG-PS | **0/1** | 15 (53.6)/13 (46.4) |
| Smoking status | **Current/former/non** | 12 (42.9)/9 (32.1)/7 (25.0) |
| Stage | **Advanced/postoperative recurrence** | 19 (67.9)/ 9 (32.1) |
| Pathology | **Adenocarcinoma/Squamous cell carcinoma** | 16 (57.1)/12 (42.9) |
| EGFR status | **Mutant type/wild type or not evaluated** | 7/21 |
| Location of mutant EGFR gene | **Ex18/ Ex19/Ex20/Ex21** | 1/2/2/2 |
| PD-L1 expression (%) | **>50/1-49/<1/unknown** | 10 (35.7)/10 (35.7)/3 (10.8)/5 (17.8) |
| ICI | **Monotherapy** | 24 (11/3/7/3) (85.7) |
|  | **Combination therapy** | 4 (14.3) |
| Line | **1/2/3/4/>5** | 10(35.7)/10 (35.7)/6 (21.4)/1 (3.6)/1 (3.6) |
| Cycles |  | 20 (3-81) |
| Response | **CR/PR/SD/PD** | 0 (0.0)/17 (60.7)/ 2 (7.2)/ 9 (32.1) |

*“advanced” means “unresectable stage III and stage IV”
