# Supplementary figures and images for: Gut microbiota diversity and specific composition during immunotherapy in responders with non-small cell lung cancer
Source: Front Mol Biosci. 2022 Oct 24;9:1040424. doi: 10.3389/fmolb.2022.1040424 (PMC9638091; doi:10.3389/fmolb.2022.1040424)

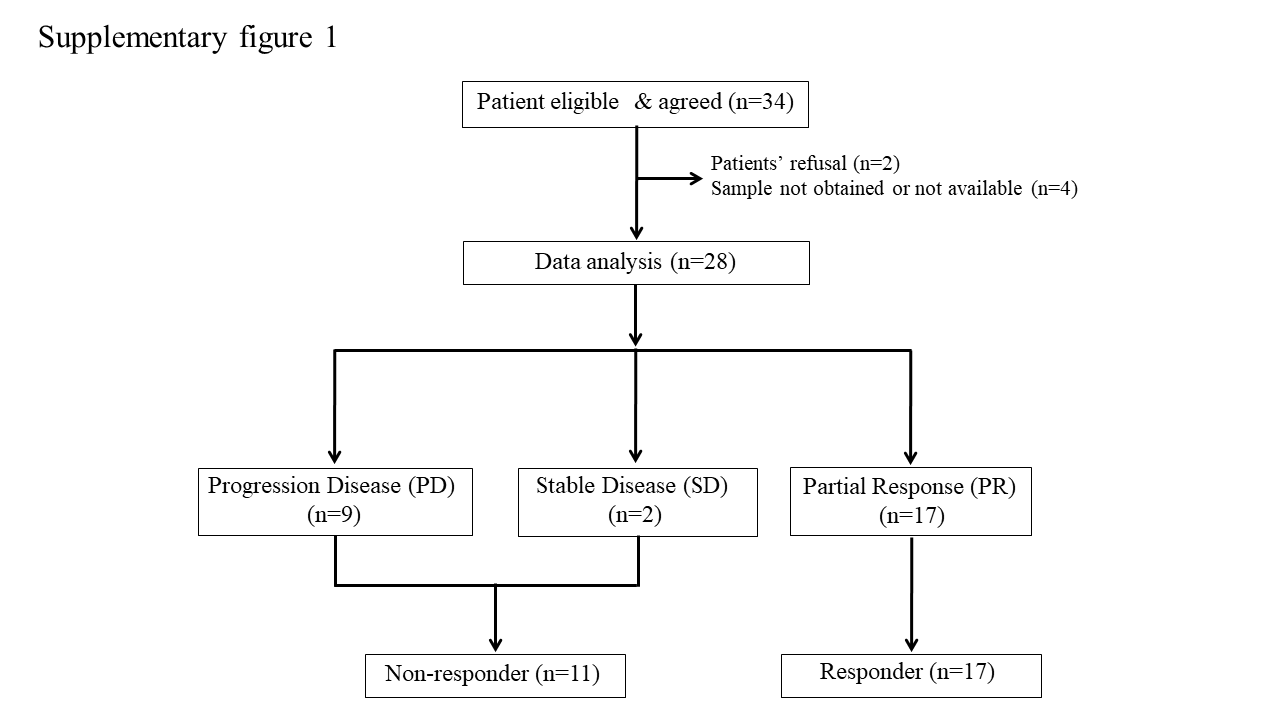

Supplement: Supplementary file 2 [file Image1.tif]
