# Supplementary material for: Gut microbiota diversity and specific composition during immunotherapy in responders with non-small cell lung cancer
Source: Front Mol Biosci. 2022 Oct 24;9:1040424. doi: 10.3389/fmolb.2022.1040424 (PMC9638091; doi:10.3389/fmolb.2022.1040424)
Supplement: Supplementary file 3 [file Table2.docx]

**Supplementary table 2**. Multivariate analyses of clinic-pathological features associated with ICI response in 28 NSCLC patients (multivariate logistic regression modelling)

| Variable | Total (n=28) |
| --- | --- |
|  | OR (95% CI), *P* value |
| Sex (male vs. female) | 3.241 (0.162- 65.034), 0.4422 |
| Smoking status (cur/for vs. never) | 0.826 (0.088- 7.769), 0.8674 |
| No. of prior systemic therapy (≤1 vs. ≥2) | 3.985 (0.194- 81.947), 0.3702 |
| Regimen (mono therapy vs. combined therapy) | 1.312 (0.063- 27.164), 0.8605 |
| Histology (Adenocarcinoma vs. Squamous cell carcinoma) | 2.306 (0.216- 24.612), 0.4891 |
| TPS ≥50% (No vs. yes) | 4.836 (0.559- 41.823), 0.1522 |

CI: confidence interval; Cur/for: current/former smoker; ICIs: immune checkpoint inhibitors; NSCLC: non-small cell lung cancer; OR: odd ratio (response vs. non-response);TPS: tumor proportion score.
